# The dry powder formulation of mixed cross-linked dextran microspheres and tetanus toxoid-loaded trimethyl chitosan nanospheres as a potent adjuvant for nasal delivery system 

**DOI:** 10.22038/ijbms.2020.49486.11313

**Published:** 2021-01

**Authors:** Mona Kabiri, Haleh Bolourian, Solmaz Dehghan, Mohsen Tafaghodi

**Affiliations:** 1 Nanotechnology Research Center, Pharmaceutical Technology Institute, Mashhad University of Medical Sciences, Mashhad, Iran; 2 School of Pharmacy, Mashhad University of Medical Sciences, Mashhad, Iran; 3 Clinical Research Development Unit, Ghaem Hospital, Mashhad University of Medical Sciences, Mashhad, Iran; 4 Biotechnology Center, Gilead Sciences, Foster City, USA

**Keywords:** Cross-linked dextran – microspheres, Mucosal immunity, Nasal immunization, Systemic responses, Tetanus toxoid, Trimethyl chitosan - nanoparticles

## Abstract

**Objective(s):**

The present study aimed to determine the immunoadjuvant efficacy of mixed cross-linked dextran microspheres (CDM) and tetanus toxoid (TT)-loaded trimethyl chitosan (TMC) nanospheres in dry powder form.

**Materials and Methods:**

The TMC nanoparticles (NPs) containing TT were produced using the ionic gelation method. Co-administration of TT-loaded TMC NPs and CDM as an absorption enhancer was performed to improve immunity against the antigen. Dry powder formulations were delivered via the nasal route in a rabbit model.

**Results:**

Among immunization groups, mixing of CDM with TT encapsulated in TMC NPs could elicit the highest titer of systemic IgG antibody. Furthermore, the addition of CDM to TT-loaded TMC enhanced the sIgA response relative to the TT solution.

**Conclusion:**

The TMC NPs had a considerable effect on mucosal and systemic immunity against the TT antigen. Therefore, the CDM excipient can be utilized for nasal immunization to elevate systemic and mucosal responses.

## Introduction

Most of the present vaccines are developed to deliver the formulation in a systemic manner such as intravenous (IV), intramuscular (IM), subcutaneous (SC), or intradermal (ID). The current vaccine formulations are administered parenterally to promote cellular and humoral immunity against pathogens. The parenteral vaccine cannot contact with a mucosal barrier to induce mucosal immunity due to the functional difference between the mucosal and systemic immune systems. On the other hand, the administration of vaccines via nasal route stimulates systemic immunity and robust mucosal immune response (1-3). Therefore, the nasal administration is applied as an alternative approach to parenteral injections to deliver peptides, proteins, and drugs. The nasal delivery of vaccines has many advantages such as low enzymatic activity, high vascularity, and effective absorption via the surface of the nasal cavity and a porous endothelial membrane. Additionally, the storage, distribution, and mass vaccination of dry powder formulations by nasal delivery are simple and economical compared to liquid vaccines (3-6). The vaccine formulations delivered by nasal route contact with nasal mucosa as the first site of interaction between antigen and host, as well as the nasal-associated lymphoid tissue (NALT) of the nasal cavity, which is essential to defend mucosal surfaces against pathogens. NALT contains the microfold-cells (M-cells), T-cell, B-cell, macrophages, dendritic cells (DCs), and other lymphoid cells to elicit and regulate mucosal immune response against inhaled antigens (7-9). 

Chitosan (CHT) is a non-toxic and hydrophilic cationic polysaccharide chemically derived by the partial deacetylation of chitin with biodegradable and biocompatible properties. CHT nanoparticles (NPs) and derivatives have potential applications as the controlled release drug and antigen delivery systems (10-12). CHT NPs can enhance membrane permeability and bioavailability of macromolecules. According to the immunohistological investigations, CHT can open the tight junctions of epithelial cells to transit across the mucosal barrier (13-16). Additionally, the electrostatic interactions between positive charges of CHT and negative charges of cell membranes or mucus could induce systemic and mucosal immunity against antigens (17, 18). The nasal perfusion and pulse-chase studies illustrated that CHT had no effect on the cellular or membrane damage in mouse models. The muco-adhesive and paracellular transport effects of CHT and its derivatives are significant parameters for the efficient delivery of vaccines by intranasal administration (12, 19-22). 

The N-trimethyl CHT (TMC) is a quaternized CHT derivative that improves physicochemical properties such as water solubility in a broader pH range relative to the CHT polymer (23, 24). The quaternization degree of TMC is dependent on the synthesized conditions and duration of the reactions (25-27). TMC particles as the potent absorption enhancer for the peptides and proteins can facilitate the paracellular diffusion of peptides due to opening the tight junctions of epithelial cells. Based on previous investigations, TMC has appropriate muco-adhesive properties that elevate the transport of peptides and proteins relative to the CHT (19, 28-33). TMC has been demonstrated to be a proper delivery system for the intranasal administration of vaccine formulations. Different approaches have been utilized to enhance the mucosal uptake of inhaled antigens including the usage of cross-linked dextran microspheres (CDM) with absorption enhancing properties to improve the immunogenicity and efficacy of mucosal vaccines (34-37). 

The purpose of this investigation was to assess the immune efficacy of encapsulated tetanus toxoid (TT) antigen as a model antigen in TMC NPs with CDM as a penetration enhancer to induce the mucosal and systemic responses followed by the nasal administration in a rabbit model. 

## Materials and Methods

***Materials***

The alum-adsorbed TT (50 Lf/m) and TT (2750 Lf/ml) solutions were provided by Razi Institute (Iran). CHT (MW 120 kDa, and deacetylation degree 93%) was purchased from Primex (Norway) to synthesize the TMC polymer. Additionally, CDM (Sephadex G-150) was obtained from Biogen (Sweden). Tripolyphosphate (TPP), Lactose, Span 80, and Tween 80 were used from the Merck group (Germany). Anti-rabbit secretory immunoglobulin A (sIgA) and immunoglobulin G (IgG) antibodies were purchased from Bethyl Laboratories Inc. (USA) and Sigma company (USA), respectively. Coomassie Brilliant Blue was obtained from Fluka (Switzerland). The Micro BCA assay kit was purchased from Pierce (Thermo Fisher Scientific, USA) and all other chemicals used were of analytical grade. 

***Synthesis and characteristics of TMC polymer***

TMC polymer was synthesized using the methylation of CHT through the two-step procedure as described previously by Sieval *et al*, 1998 (38). The obtained TMC was dialyzed against distilled water at 4 °C for 3 days followed by lyophilizing at -54 °C (Heto, Denmark). The purified TMC polymer was characterized using nuclear magnetic resonance (NMR) in deionized water at 80 °C (25, 38). The degree of quaternization (DQ) was determined according to the follows:

DQ (%)=[[(CH_3_)_3_]/[H] × 1/9 ] × 100

 (1)

[(CH_3_)_3_] is the integral of the trimethyl amino group at 3.3 ppm and [H] is the integral of the 1 H peaks of TMC polymer between 4.7 and 5.7 ppm (38).

***Preparation and characterization of TMC nanoparticles***

TMC NPs containing TT were produced by the ionic gelation method (25). Briefly, the TMC solution (2 mg/ml) with 1% Tween 80 (w/v) was prepared in distilled water. Afterward, the TPP solution (1 mg/ml) was added dropwise to the TMC solution until the appearance of turbidity. TT-loaded TMC nanospheres were produced via dissolving TT antigen in the polymer solution (120 Lf of TT in 5 ml TMC), followed by adding the TPP solution. Nanospheres were centrifuged at 10,000 g for 15 min and pellets were resuspended in 10 mM phosphate-buffered saline (PBS) at pH 7.4. The particle size and surface charge of TMC NPs were measured using photon correlation spectroscopy by Zetasizer (Nano ZS, Malvern Instruments, UK). The experiments were accomplished at least in triplicates. 

***SDS-PAGE analysis of TT entrapped in TMC nanospheres***

The integrity of TT-loaded TMC NPs was analyzed using sodium dodecyl sulfate-polyacrylamide gel electrophoresis (SDS-PAGE) technique. All samples were loaded on a 10% acrylamide gel and the silver nitrate staining was used to visualize the bands of protein.

***Loading efficiency of TT antigen in TMC nanoparticles***

The TT-loaded TMC was identified from the difference between the free and total amounts of TT in the supernatant followed by centrifugation at 15,000 g for 20 min at 10 °C. The BCA assay was applied to obtain the concentration of non-entrapped TT antigen in TMC NPs and the suspension of empty TMC NPs was utilized as a blank. The loading efficiency (LE) was determined by the following equation: 

$\mathrm{LE}(\mathrm{\%0}=\frac{\mathrm{The total amount of TT}-\mathrm{free TT antigen}}{\mathrm{The total amount of TT}}\times 100$

 (2) 

Additionally, the yield of TMC NPs and also TT loading content (LC) were calculated as follows:

$\mathrm{Yield}(\%)=\frac{\mathrm{Weight of TMC nanosphers}}{\mathrm{Weight of feeding polymer}+\mathrm{TT antigen}}\times 100$

(3)

$\mathrm{LC}\left(\%\right)=\frac{\mathrm{The amount of TT in TMC NPs}}{\mathrm{TMC NPs dry weight}}\times 100$

 (4) 

***Release assay***

The nasal release of TT antigen from TMC NPs was performed using the vertical Franz diffusion cell, to simulate the humidity of the nasal cavity. The mentioned diffusion cell is comprised of two compartments with a membrane fixed between the donor and acceptor chambers. The acceptor chamber was filled with 20 ml of fresh PBS solution at pH 7.4, which stirred by a magnetic stirring bar. The nanospheres (25 mg) were poured evenly across the hydrated filter paper mounted in the diffusion cell, which was in contact with the buffer of the acceptor compartment. The release experiments were carried out at 37 °C for 4 hr. At regular time intervals, 400 µl of each sample was withdrawn from the acceptor chamber and replaced with a fresh PBS. The concentration of released TT antigen from TMC nanospheres was measured using the Micro BCA protein assay method. All experiments were conducted in triplicates. 

***Immunization studies***

White albino rabbits weighing between 2 to 2.5 kg were supplied by Pasteur Institute (Iran) to accomplish the *in vivo* study. All animal experiments were performed according to the Mashhad University of Medical Sciences Ethical Committee Acts, which complied with the ARRIVE guidelines and also conducted under the National Institutes of Health (NIH) guide for the care and use of laboratory animals. Rabbits were immunized in groups of four, three times at days 0, 14, and 28 (two weeks interval) via nasal administration as the following formulations: 40 Lf TT antigen solution (TT); 40 Lf TT antigen in TMC + CDM (TMC (TT) + CDM); and 40 Lf TT antigen in TMC + lactose (TMC (TT) + Lac). Additionally, intramuscular administration of the alum-adsorbed TT vaccine (Alum TT) was performed as a positive control to compare the efficacy of vaccine formulations. 

Rabbits were sedated with ketamine hydrochloride) 40 mg/kg (using intramuscular injection to prevent sneezing during nasal administration. Animals were immunized with nanospheres powder (5 mg) or TT antigen solution (100 µl) by the intranasal route to assess the immune responses against the TT antigen. For the nasal delivery of dry powder formulations, powders were poured in polyethylene tubes (2.0 mm diameter) and linked to a syringe. The mentioned tubes were placed in an animal nose (0.5 cm) and 10 ml of air was injected into the tube. The inoculated animals were bled two weeks after the last booster to collect sera for antibody assays. Subsequently, rabbits were sacrificed and the nasal cavity was rinsed with PBS (5 ml, pH 7.4). All samples were kept at -70 °C until immunological assays.

***Determination of antibody responses***

The serum sample of each rabbit was collected to evaluate the total IgG antibody. Additionally, the nasal lavage samples from the nasal cavity of animals were used for the sIgA antibody assay. TT-specific antibody responses in the sera and nasal lavages were determined by end-point titration using an enzyme-linked immunosorbent assay (ELISA) as described previously (39). Accordingly, the serum and nasal lavage samples of unimmunized rabbits were utilized as negative control. End-point dilution titers for total sIgA and IgG antibodies were detected as the highest dilution with absorbance values (OD=450 nm) equivalent to the negative control.

***Statistical analysis***

Immunization data were performed by one-way ANOVA using GraphPad Prism (version 7.0) to assess the significance of the differences between formulations followed by the *post ho*c Tukey-Kramer test. The results are presented as the mean (*n* = 4) ± standard deviation (SD). The *P*-values of less than 0.05 were considered significant.

## Results

***Characteristics of synthesized TMC by NMR spectroscopy***

The analysis of NMR spectra revealed that a reproducible degree of quaternization was about 24%, followed by synthesizing TMC polymer using the two-step methylation procedure (data not shown). 

***Preparation and characteristics of TMC nanospheres***

The TMC NPs were produced using ionotropic gelation of TMC polymer with the TPP solution. According to this method, the negatively charged TPP solution can interact with the positive charges of CHT (amino groups) by electrostatic interactions. The mentioned method has several advantages including the absence of heat and organic solvent during the preparation of TMC NPs. The mean intensity diameters of TMC NPs (n=5) without or with TT antigen was 378.13±50.9 nm and 447.3±58.07 nm, respectively. Additionally, the zeta potential of TMC NPs in the absence or presence of TT antigen was 3.32±0.05 mV and 2.28±0.07 (n= 5), respectively.

***Determination of the protein stability***

**Figure 1:**
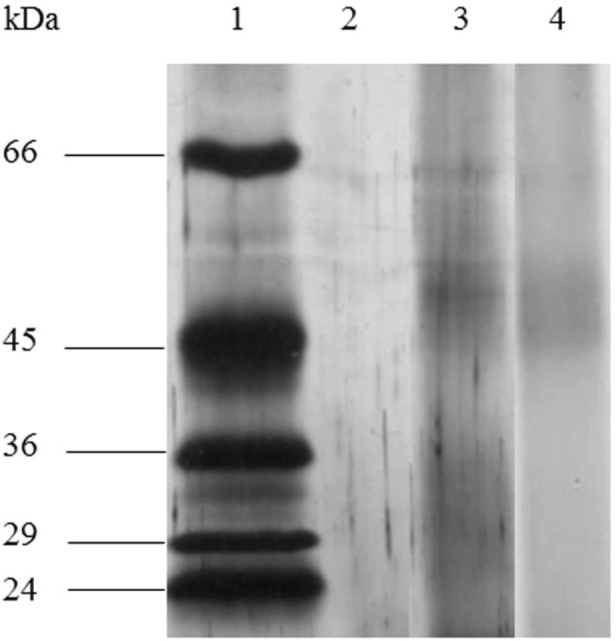
The SDS-PAGE profile of tetanus toxoid (TT) antigen and TT-loaded trimethyl chitosan (TMC) nanospheres. Lane 1: protein molecular weight marker, lane 2: blank TMC, lane 3: TMC containing TT antigen, lane 4: antigen solution

The SDS-PAGE analysis was utilized to assess the effect of the TMC preparation procedure on TT antigen integrity. As shown in Figure 1, the identical bands were detected for both TT and TT-loaded TMC NPs. This result revealed the stability of the TT structure in TMC NPs during the preparation and encapsulation process. 

***Loading efficiency of TT antigen in TMC nanospheres***

The loading efficiency of TT in TMC NPs was calculated to be 43.25%±3.56 (n=5). The yield of TMC NPs and TT loading content was determined as 86%± 23.4 and 90%±27.1, respectively.

***The release study of TT from TMC nanoparticles***

**Figure 2:**
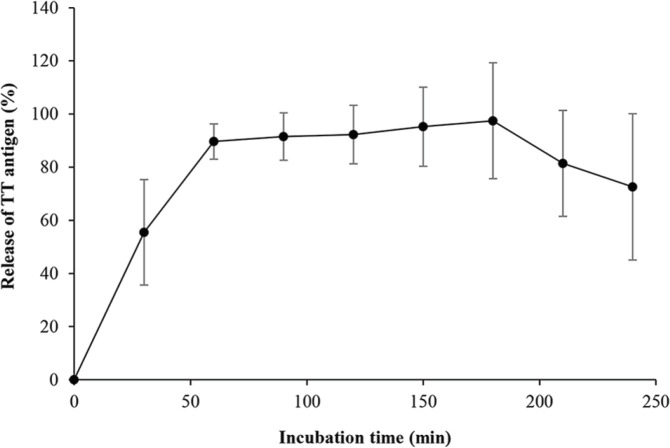
The release profile of entrapped tetanus toxoid (TT) in trimethyl chitosan (TMC) nanospheres. Samples were withdrawn from the acceptor chamber and replaced with phosphate-buffered saline at 30 min time intervals for 4 hr. Data points represent the mean±SD of triplicate samples

The release assay of the TT antigen was accomplished in a diffusion chamber to mimic the humid environment of the nasal mucosa (Figure 2). The nasal release experiments of TT from TMC NPs demonstrated a burst release of 55.47%±19.83 within 30 min and reached 72.6%±27.52 in 4 hr. 

***Anti-TT IgG titers***

**Figure 3:**
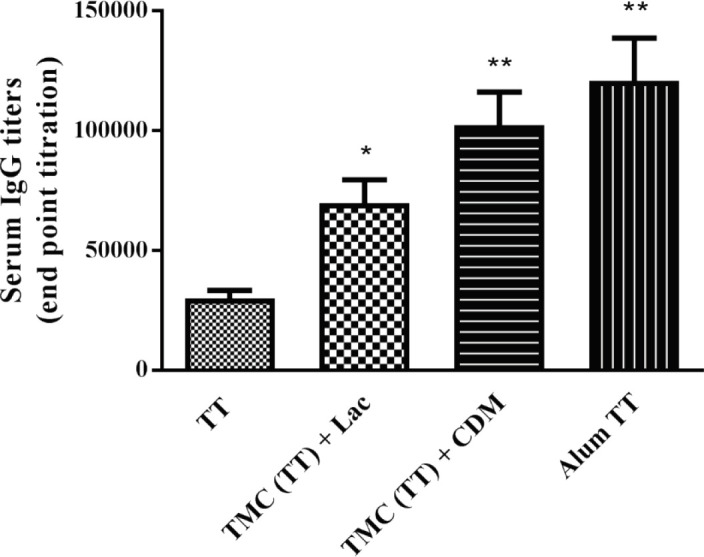
The serum anti-tetanus toxoid (TT) immunoglobulin G titers of immunized rabbits with different vaccine formulations by nasal administration. Animals were inoculated at days 0, 14, and 28 with one of the following formulations: TT solution; trimethyl chitosan (TMC) containing TT+cross-linked dextran microspheres (CDM); and TMC (TT)+Lactose (Lac). The intramuscular injection of Alum TT was administrated as a positive control. At days 42, sera were collected to identify the IgG titers by end-point titration using the ELISA technique. Results present the mean±SD (n=4)

The serum IgG titers of immunized rabbits with various vaccine formulations were obtained via the end-point titration using an ELISA method (Figure 3). The titers of IgG antibody were significantly higher in inoculated rabbits with TMC (TT) + CDM, TMC (TT) + Lac, and Alum TT in comparison with the TT solution. The highest titer of IgG (*P*<0.05) was observed in the rabbits received Alum TT and TMC (TT) + CDM formulations through IM or nasal administration, respectively. Moreover, no significant difference (*P*>0.05) was determined between vaccinated animals by TMC (TT) + CDM and Alum TT vaccines. 

***Anti-TT sIgA titers***

**Figure 4:**
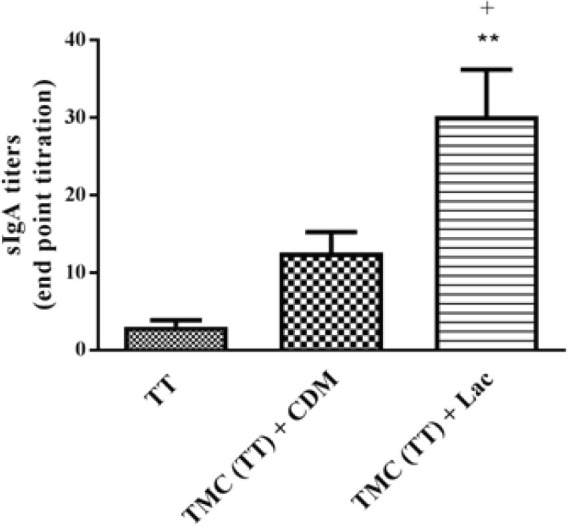
The nasal lavage anti-tetanus toxoid (TT) immunoglobulin A titers of vaccinated rabbits followed by nasal delivery of formulations. Rabbits were immunized at days 0, 14, and 28 with one of the following formulations: TT solution; trimethyl chitosan (TMC) containing TT + cross-linked dextran microspheres (CDM);+ cross-linked dextran microspheres (CDM); and TMC (TT) + Lactose (Lac). At days 42, the nasal lavages were collected to determine the sIgA titers by end-point titration using the ELISA method. Data were expressed as the mean±SD (n=4)

The sIgA titers were identified from nasal lavage of immunized rabbits using the end-point titration via an ELISA method. As shown in Figure 4, the highest sIgA titers were found in inoculated rabbits with TMC NPs formulations including TMC (TT) + Lac and TMC (TT) + CDM. The titer of sIgA antibody was significantly (*P*< 0.01) higher for nasal administration of TMC (TT) + Lac vaccine compared to TMC (TT) + CDM or TT solution. The lowest sIgA titer was obtained in the group received the TT solution by the nasal route. 

*Significant difference between TT solution and test groups *(*P*<0.05), **(*P*<0.01), and ***(*P*<0.001)

*Significant difference between TT solution and test groups *(*P*<0.05), **(*P*<0.01), and ***(*P*<0.001)

+Significant difference between test groups

+(*P*<0.05), ++(*P*<0.01), and +++(*P*<0.001)

## Discussion

Dry powder forms of particulate delivery systems with muco-adhesive properties including CHT and its derivatives were developed as appropriate candidates for mucosal immunization. The purpose of our investigation was to evaluate the immune efficacy of synthesized TMC NPs as an antigen delivery system for nasal delivery of TT antigen in the presence of CDM or lactose excipient. The co-administration of lactose and TT-loaded TMC NPs were accomplished to promote the inhalation efficacy of the vaccine formulation. Additionally, the CDM adjuvant was physically mixed with TMC NPs loaded with TT to elevate the immunogenicity and efficiency of vaccine formulations. The dry powder formulations were delivered via the nasal route in a rabbit model to determine the titers of sIgA and IgG antibodies against the TT antigen. The dry powder form of vaccine formulations has many advantages including more microbiological and chemical stability, easier storage without cold chain, mass vaccination, and better distribution as well as more affordable compared to liquid formulations (23, 35, 36, 40, 41).

The mean size of synthesized TMC NPs without or with TT antigen was about 378 nm and 447 nm, respectively that is expected to interact efficiently with DCs. Similar to the viruses, the NP formulations with a size less than 500 nm can be quickly recognized by antigen-presenting cells (APCs) during processing and presentation of antigen followed by strongly uptake by DCs to evoke the immune response against pathogens. In contrast, the particle formulations with a size range of 0.5 to 5 µm are effectively uptaken by macrophages (42-45). Additionally, the surface charge of NPs is a crucial factor to induce potent immunity. Previous investigations illustrated that the positively charged particles enhanced the effective interaction with DCs or macrophages (42, 44, 46-48). The cationic surface of TMC polymer could elicit higher uptake of TMC particles by APCs due to the electrostatic interactions with the negative charge of cell membranes relative to anionic particles. 

The nasal release study of TT-loaded TMC NPs was performed in a diffusion chamber to simulate the temperature and humid environment of the nasal cavity. Therefore, to assess the release profile of the TT antigen, the nanospheres were in contact with a wet and warm membrane similar to the nasal mucosa. Based on the release profile, the TT antigen demonstrated a burst release within 1 hr, suggesting that most of the TT was released from TMC NPs followed by a plateau release up to 4 hr. Previous studies revealed that the half-life of clearance is approximately 15 to 20 min in a nasal cavity of humans (36, 49, 50). The particulate delivery system including CHT and derivatives protects antigens from proteolytic enzymes and promotes the immunogenicity and efficacy of nasal vaccines (51, 52). Therefore, the TMC NPs as the particulate delivery system with muco-adhesive effects could increase the maintenance of vaccine formulation in the nasal mucosa. 

According to the antibody assays, the incorporation of TT into TMC NPs could significantly enhance the titers of sIgA and IgG antibodies in rabbits vaccinated through the nasal route compared to the antigen alone. The sIgA antibody indicates mucosal immunity against pathogens, whereas the systemic humoral immune response is mediated by IgG antibody (53). Our results determined that the nasal administration of TMC (TT) formulation in the presence of CDM or lactose excipient could elicit robust systemic humoral and mucosal responses compared to the TT solution. 

Following the intranasal delivery of TMC formulations, the higher titer of mucosal IgA antibody was observed for TMC (TT) + CDM and TMC (TT) + Lac vaccines than the TT solution. The TT-loaded TMC NPs in the presence of lactose excipient could considerably enhance the sIgA titer indicating a potent mucosal immune response. Based on our results, TMC formulations could pass through the NALT in the respiratory tract to interact with mucosal inductive tissues such as the Peyer’s patches. Subsequently, the TT-loaded NPs could be efficiently uptaken by the M-cells located in the mucosal inductive tissues to secrete the mucosal sIgA antibody and induced robust mucosal immunity (4, 7, 54, 55). The results of the sIgA test confirmed that the encapsulation of TT into TMC NPs with muco-adhesive properties could promote the maintenance of formulations in the nasal cavity to interact effectively with M-cells and other mucosal immune cells and elicited high mucosal immunity. It has been proved that antigen molecules in the particulate form are more uptaken by microfold cells relative to antigen solution (6, 34, 52, 54, 56). 

Although the TMC (TT) + CDM vaccine induced the higher titer of the sIgA antibody compared to the TT solution, no significant difference was found between the mentioned formulations. Some of our previous studies illustrated that the mixture of CDM with PLGA NPs or alginate microspheres loaded with TT could enhance the titers of the sIgA antibody followed by nasal administration (35, 41). However, many investigations indicated that the co-administration of CDM with TT antigen via nasal route had no considerable effect to evoke the mucosal sIgA responses (36, 57, 58). Tabassi *et al.* reported that the addition of CDM to TT antigen could induce the lower mucosal sIgA titer in comparison with the TT solution, while the high mucosal immunity was stimulated by the TT solution. This study demonstrated that the lower titers of the sIgA antibody elicited with CDM + TT formulation might be due to the size of microspheres and their inability to interact with the M-cells of NALT (57). Additionally, the nasal delivery of TT-loaded CDM and also co-administration of TT with CDM in the absence or presence of the saponin adjuvant could not significantly increase nasal sIgA titers relative to the TT solution (58). According to this study, the administration of CDM with particulate delivery systems may elevate the systemic immune responses, whereas the mentioned formulation has no considerable effect on mucosal immunity. In another investigation, the mixing of CDM with TT-loaded CHT NPs demonstrated the lower mucosal sIgA titer compared to (CHT) TT + Lac vaccine. The results revealed that the encapsulated TT in CHT NPs could significantly elicit a high level of sIgA response against TT antigen (36). 

Although the co-administration of CDM or lactose excipient with TMC (TT) formulation could induce robust systemic immunity against TT, the intranasal delivery of TMC (TT) + CDM formulation was more effective to elicit the high titer of IgG antibody compared to TMC (TT) + Lac vaccine. Additionally, no significant difference was found between the nasal delivery of TMC (TT) + CDM and IM injection of the Alum TT vaccine. Accordingly, the addition of CDM to TT-loaded NPs has an effective role to produce a high level of systemic IgG antibody response. The CDM has been reported as a safe absorption enhancer with adjuvant effect to improve the epithelial absorption and permeation of various antigens due to opening the intercellular tight junctions (36, 37, 57-59). Chandler *et al. *demonstrated that the co-administration of insulin and diethylaminoethyl-dextran (DEAE-Dextran) promoted the nasal absorption of insulin formulation in a mouse model (59). Based on our previous studies, CDM could considerably improve the mucosal and systemic immune responses (35, 41, 58). The addition of CDM as the dried hydrogel particles to co-encapsulated TT antigen and saponin adjuvant in alginate microparticles elicited the high systemic IgG titers (41). The co-administration of CDM with TT-loaded PLGA (poly [lactide-co-glycolide]) NPs illustrated the high level of IgG production relative to the PLGA (TT) + lactose or TT solution (35). The co-delivery of TT and saponin by cross-linked dextran microspheres could significantly elevate IgG titers compared to the mixing of TT with CDM or saponin (58). Moreover, the mixture of CDM with TT-loaded CHT NPs induced a high titer of IgG antibody in comparison with the TT solution (36, 58). 

Taken together, the co-administration of CDM with antigen delivery systems such as TMC nanospheres can induce potent systemic immune responses, whereas CDM has no significant effect to elicit mucosal sIgA response. 

## Conclusion

The utilization of particulate delivery systems was developed to overcome the enzymatic degradation, rapid mucociliary clearance, and low absorption of antigens and drugs following nasal administration. The dry powder form of TT-loaded TMC NPs with or without CDM adjuvant was evaluated after intranasal immunization in a rabbit model. The encapsulated TT in TMC NPs induced high sIgA and IgG titers indicating the robust mucosal and systemic responses against TT antigen, respectively. The addition of CDM powder to TMC vaccine formulation could significantly promote the systemic IgG responses compared to TMC (TT) in the presence of lactose. Although, the TMC formulation with CDM increased the mucosal sIgA titer, no considerable difference was observed between the mentioned vaccine formulation and TT solution. According to our study, CDM excipient can be used as an efficient adjuvant to induce potent systemic responses. 
